# Simple yet effective heuristic community detection with graph convolution network

**DOI:** 10.1038/s41598-025-22860-z

**Published:** 2025-11-10

**Authors:** Hong Wang, Yinglong Zhang, Zhangqi Zhao, Zhicong Cai, Xuewen Xia, Xing Xu

**Affiliations:** College of Physics and Information Engineering, Minnan Normal University, Zhangzhou, 363000 Fujian China

**Keywords:** Graph convolution network, Adaptive community detection, Node-community relationship modeling, Soft modularity optimization, Computer science, Information technology

## Abstract

In recent years, graph neural network-based community detection methods have integrated local structure and node attributes, incorporating various optimization strategies with notable progress. However, most current algorithms require predefining the number of communities, introducing human bias, and rely on contrastive objectives or data augmentation, leading to extra hyperparameters and complexity. To address these issues without sacrificing detection quality, we propose an adaptive community detection framework that eliminates contrastive learning and the need for pre-specified community numbers, simplifying training and reducing prior dependency. First, the adaptive detection method is introduced to ensure the identification of high-quality structural communities as reliable global references. Then, a novel mechanism for modeling node-community relationships is proposed, integrating global structure, local structure, and attribute information into a unified space. Finally, a reconstructed soft modularity loss is applied to optimize node-community relationships end-to-end, enhancing community structure without data augmentation or contrastive learning. The proposed approach is efficient to train and computationally lightweight, demonstrating superior detection efficiency and competitive accuracy across multiple graph datasets compared to traditional and recent deep learning methods. The code is available at https://github.com/wuanghoong/Less-is-More.git.

## Introduction

Numerous complex systems in the real world are composed of interconnected entities. To uncover their underlying principles, such systems are often abstracted as complex network models^1^, including social networks (Facebook^2^, Twitter^3^), computer networks (the Internet, LAN^4^), and biological networks (gene regulatory networks^5^, protein-protein interaction networks^6^), among others. In a complex network, entities are represented as nodes, and the relationships between them correspond to edges. A set of nodes with dense internal connections often shares similar attributes or functions in reality. Such node clusters are referred to as “communities”. Communities exhibit cohesion and homogeneity internally, while connections between different communities are sparse and heterogeneous. Therefore, community detection has become a crucial approach for analyzing complex network structures and uncovering group behaviors and functional patterns.

Community detection aims to identify clusters of nodes within a network that are densely interconnected and share similar attributes, known as community structures, which are characterized by dense internal links and sparser connections between communities^7^. Traditional community detection methods primarily include techniques such as modularity optimization, spectral clustering, random walks, and label propagation^8^. While effective in small to medium-sized networks, these methods reveal significant limitations when applied to today’s large-scale complex networks. Many traditional approaches rely solely on topological structure, resulting in high computational complexity that hinders scalability. Moreover, they often overlook rich node attributes, limiting their ability to capture complex relational patterns in real-world networks.

In recent years, deep learning methods have emerged as a new research focus in community detection, leveraging strong nonlinear representation capabilities and efficient large-scale data processing^9^. The application of deep learning in this field has evolved from graph embedding to graph neural networks (GNNs). Early graph embedding methods^10–13^ mapped network nodes into low-dimensional vectors so that structurally similar nodes are close in the embedding space, after which standard clustering algorithms^14,15^ were applied to group these vectors. However, the inherent disconnection between the embedding and clustering stages in this two-step paradigm motivated the shift toward end-to-end GNN frameworks, which have become the mainstream architecture for community detection. Typical GNN models offer diverse information aggregation schemes for community detection. As a foundational work, GCN (Graph Convolutional Network)^16^ employs spectral graph convolution to aggregate neighborhood features, yet its equal-weight aggregation struggles to distinguish the importance of different neighbors. GAT (Graph Attention Network)^17^ addresses this by introducing an attention mechanism, enabling dynamic weighting of neighbor contributions. In contrast, GAE (Graph Autoencoder)^18^ adopts an unsupervised approach, using an encoder (e.g., GCN) to learn node representations and a decoder to reconstruct the adjacency matrix, which helps preserve community structure in low-dimensional embeddings.

Building on these classic models, researchers have integrated advanced learning strategies with GNN architectures to enhance the perception and utilization of complex network information^19^. For instance, by designing contrastive tasks^20–26^ at the node, subgraph, or community level, GNNs can learn intrinsic structural patterns without relying on ground-truth labels, enabling the identification of stable community features across varying augmented views. Another direction involves guiding the optimization process through differentiable objective functions^27–32^. For example, some methods allow GNNs to jointly optimize both representation learning and community partitioning in an end-to-end manner, by transforming modularity maximization into a differentiable loss function^33^. These strategies not only expand the expressive power of GNNs in community detection but also promote the development of more adaptive, interpretable, and integrated solutions.

Despite significant progress in GNN-based community detection, overall performance and practical utility remain constrained by several core issues. First, there is an excessive reliance on specific learning strategies: current methods heavily depend on carefully designed graph data augmentation or contrastive learning mechanisms. Their performance is sensitive to the choice of augmentation strategies and the quality of sample pairs, lacking inherent robustness. Second, the optimization process is often overcomplicated. Many advanced models enhance performance by stacking multiple loss functions or introducing complex contrastive mechanisms, resulting in bloated model architectures, increased training difficulty, and challenges in convergence. Finally, adaptive capability remains insufficient: although end-to-end learning has become mainstream, practical GNN models that can fully adaptively determine the number of communities without any prior knowledge are still relatively scarce.

To address the above challenges, the main contributions of this paper are summarized as follows:

1. Adaptive Detection: By leveraging the Louvain algorithm combined with a size-based filtering strategy, the method adaptively extracts high-quality structural communities without pre-defined cluster numbers to provide reliable global structural information.
2. Global-Local-Attribute Integrated Node-Community Relationship Modeling: Within a unified shared embedding space, a single-layer GCN integrates local topology and node attributes to generate node representations, while structural communities are projected as structural center vectors. A soft relational matrix, constructed based on node-center similarity, serves as the optimization objective for community refinement and interpretation.
3. Reconstructed Soft Modularity Objective Based on Node-Community Relations: Using the soft relational matrix, a reconstructed soft modularity loss is formulated, enabling end-to-end optimization of node-community affiliation probabilities without relying on contrastive learning or data augmentation. This results in a more streamlined and robust training process.
4. Efficiency and Practical Applicability: The framework is lightweight and easy to train. Experiments on multiple real-world network datasets demonstrate higher detection efficiency and competitive accuracy compared to both traditional and recent deep learning approaches.

## Related work

### GNN-based community detection

GNN-based community detection methods learn node embeddings to identify community structures. These approaches leverage the powerful capability of GNNs in modeling graph data, incorporating both topological information and node features to significantly improve detection performance. Compared to traditional methods, GNN-based techniques can more accurately capture complex community patterns, particularly in graphs with rich node attributes. Recent studies have integrated various learning strategies to optimize node representations, thereby enhancing the accuracy and robustness of community detection. These methods not only strengthen the modeling of graph structure but also exploit underlying relationships among node features to further refine community partitioning. CommDGI^21^ introduces a dual objective combining “community mutual information” and modularity, coupled with a differentiable soft K-means clustering layer, enabling end-to-end joint optimization of GNN representation learning and community detection. SGCMC^22^ employs a graph attentional autoencoder with a self-supervised mechanism to co-optimize node representations and affinity matrix learning, achieving end-to-end training of multi-view GNNs for soft clustering and significantly enhancing joint modeling of graph structure and nonlinear semantics. DCGL^23^ addresses generic clustering scenarios without prior graph structures by proposing a pseudo-siamese network that parallelizes GCN and autoencoder. It applies centroid-guided contrastive loss at the feature level and local-global graph contrast at the cluster level to explicitly optimize cluster compactness, significantly improving discriminativity and robustness. DCLN^24^ incorporates a dual-level contrastive learning mechanism, introducing high-order neighborhood similarity constraints at the node level and dimension decorrelation constraints at the feature level, effectively alleviating representation collapse and enhancing structural and feature discrimination. SCGC^25^ replaces GNN with a lightweight MLP, condensing multi-hop structures into “influence scores,” and uses an augmentation-free IAC loss to dynamically guide embedding learning, achieving highly efficient and scalable deep graph clustering. CPGCL^26^ enables a GCN to simultaneously output node embeddings and community distributions, uses community probabilities to weight contrastive loss dynamically, and reinforces high-confidence samples in a self-supervised manner to suppress false negatives and co-optimize community assignment and representation.

Despite the notable progress achieved by GNN-based community detection methods, their performance often heavily relies on data augmentation or contrastive learning strategies. However, improperly designed data augmentation may disrupt the inherent semantic structure of graph data, while the effectiveness of contrastive learning is highly dependent on the quality of positive and negative sample pairs. If poorly constructed, these strategies can mislead the model into learning spurious correlations instead of essential community structures and compromise its generalization capability as a result.

### Modularity maximization

Modularity, as one of the standard metrics for evaluating the quality of network community division, primarily assesses the density of connections within communities and the sparsity of links between them^34^. In the context of modularity maximization, Ulrik Brandes^35^ demonstrated that maximizing modularity is an NP-complete problem, a finding that spurred the development of heuristic approaches such as spectral relaxation^36^ and greedy algorithms^37^. However, these earlier studies focused predominantly on network topology, often overlooking the interrelationships between node attributes. With the rapid advancement of deep learning, integrating deep learning techniques with modularity maximization has emerged as a mainstream optimization strategy in community detection, leading to more accurate and robust partitioning solutions. Yang et al.^27^ proposed a Deep Nonlinear Reconstruction (DNR) method, which uses stacked autoencoders to perform nonlinear low-dimensional embedding and reconstruction of the modularity matrix, overcoming the limitations of traditional linear methods in representation capability. Alexandre Hollocou et al.^28^ introduced a soft clustering relaxation method based on modularity maximization, along with an efficient local sparsification algorithm, allowing nodes to probabilistically belong to multiple communities. Guillaume Salha-Galvan et al.^29^ developed a modularity-aware graph autoencoder that incorporates community-preserving message passing and a modularity-inspired regularization loss, effectively integrating graph structure and community information during encoding to significantly improve detection performance. DGCluster^30^ proposed a deep graph clustering framework based on differentiable modularity maximization. By softening the modularity objective and combining it with GNN-based community similarity, it achieves efficient clustering without predefining the number of communities. MAGI^31^ reformulated modularity maximization from a contrastive learning perspective, showing its equivalence to graph contrastive learning guided by modularity coefficients, and proposed a community-aware self-supervised pretraining task that captures high-order proximity without graph augmentation. MOMCD^32^ introduced a motif-weighted modularity optimization model, integrating high-order motif structures and low-order edge information into a unified weighting scheme. By constructing a motif adjacency matrix and defining a weighted modularity metric, it uses heuristic algorithms to maximize modularity, enabling higher-quality and higher-order community detection.

The integration of deep learning with modularity not only enables effective fusion of node features and network structure from complex networks but also injects community-level semantic information into node representation learning through the objective of modularity maximization. Furthermore, end-to-end deep learning frameworks avoid the complex iterative processes of traditional optimization algorithms, significantly reducing computational overhead.

Table 1 provides a comparative summary of the aforementioned relevant algorithms and the method proposed in this paper, offering a clear overview of the design differences among them. Although the MOMCD method in Table 1 appears to be consistent with our method at first glance, there are essential differences between them. Both MOMCD and Louvain belong to traditional methods and do not take into account the attribute features of nodes.

**Table 1:** Review and comparison of related algorithms.

Model	Year	Types of GNNs	Requirement to specify the number of communities	Contrastive learning	Modularity optimization	Joint optimization
K-means^15^	1982	Without GNN	✓			
Louvain^38^	2008	Without GNN			✓	
Yang et al.^27^	2016	Stacked Auto-Encoder	✓		✓	
Alexandre Hollocou et al.^28^	2019	Without GNN			✓	
CommDGI^21^	2020	GCN	✓	✓	✓	✓
SGCMC^22^	2021	GATE		✓		✓
Guillaume Salha-Galvan et al.^29^	2022	Modularity-Aware GAE/VGAE	✓		✓	✓
DCLN^24^	2023	GCN	✓	✓		✓
DGCluster^30^	2023	GCN			✓	✓
DCGL^23^	2024	GCN and AE	✓	✓		✓
MAGI^31^	2024	GCN	✓	✓	✓	
MGCN^39^	2024	GAE and AE	✓			✓
MOMCD^32^	2025	Without GNN			✓	
SCGC^25^	2025	Without GNN	✓	✓		✓
CPGCL^26^	2025	GCN	✓	✓		✓
Our		GCN			✓	

## Preliminaries

### Definition 1

*Undirected Attributed Graph.* An undirected attributed graph can be formally defined as a triple $G = (V,E,X)$. Where $V=\{v_1, v_2, v_3, \ldots , v_n\}$ is the set of nodes, and $n = |V|$ denotes the total number of nodes in the network. $E \subseteq V \times V$ is the set of edges, representing pairwise relationships between nodes. An edge $e_{ij} = (v_i, v_j) \in E$ indicates a connection between nodes $v_i$ and $v_j$. Let $M=|E|$ denote the total number of edges. $X = \left\{ x_1, x_2, x_3, \ldots , x_n \right\} \in \mathbb {R}^{n \times d}$ is the node attribute matrix. The i-th row vector $x_i \in \mathbb {R}^d$ corresponds to the d-dimensional feature representation of node $v_i$. The topological structure of the graph is characterized by the adjacency matrix $A \in \left\{ 0, 1 \right\} ^{n \times n}$ , where $a_{ij}=1$ if and only if $\left( v_i, v_j \right) \in E$ , otherwise $a_{ij}=0$. Based on the adjacency matrix, the degree matrix $D=diag(d_1,d_2,d_3,...,d_n)$ is defined as a diagonal matrix whose elements $d_i = \sum _{j=1}^{n} a_{ij}$ represent the degree of node $v_i$, i.e., the number of neighbors directly connected to it.

### Definition 2

*Graph convolutional networks.* The core idea of GCN originates from spectral filtering of graph signals in spectral graph theory. Its layer-wise propagation rule represents a first-order Chebyshev polynomial approximation of the graph Laplacian operator, achieving efficient spatial-domain neighborhood aggregation. The propagation rule for a single-layer GCN is given by:

1 $$\begin{aligned} {Z^{(l+1)} = \text {GCN}\left( Z^{(l)}, A\right) = \sigma \left( \widetilde{A} Z^{(l)} W^{(l)}\right) } \end{aligned}$$

where $Z^{(l)} = \left\{ z_1^{(l)}, z_2^{(l)}, z_3^{(l)}, \ldots , z_n^{(l)} \right\} \in \mathbb {R}^{n \times d'}$ denotes the node representation matrix at the $l$-th layer, with the initial input $Z^{(0)} = X$. $\widetilde{A} = \hat{D}^{-\frac{1}{2}} \hat{A} \hat{D}^{-\frac{1}{2}}$ is the normalized adjacency matrix, which stabilizes the training process and mitigates gradient explosion or vanishing. $\hat{A} = A + I$ is the adjacency matrix with self-loops added, where $I$ is the identity matrix, ensuring that each node retains its own features during aggregation. $\hat{D}$ is the degree matrix of $\hat{A}$, i.e., ${\hat{D}}_{ii} = \sum _{j} {\hat{A}}_{ij}$. $W^{(l)}$ is the trainable weight parameter matrix of the l-th layer. $\sigma (\cdot )$ is the nonlinear activation function (PReLU), enhancing the model’s nonlinear expressive capacity and feature discrimination ability.

The objective of node representation learning is to obtain a low-dimensional, dense vector representation $z_i$ for each node through stacked GCN layers, which simultaneously encodes both its local topological structure and intrinsic attribute features.

### Definition 3

*Structural community centers.* The purpose of structural community centers is to learn a prototype vector for each potential community in the graph, which represents the core characteristics of that community in the feature space. Given the learned embedding matrix $H \in \mathbb {R}^{n \times d'}$ from representation learning and the pre-detected structural communities $\{ C_1, C_2, \dots , C_k \}$, the structural center $u_j$ of the j-th community can be computed by aggregating the representations of its member nodes. A common approach is to compute the mean vector:

2 $$\begin{aligned} {u_j = \frac{1}{|C_j|} \sum _{v_i \in C_j} h_i} \end{aligned}$$

where $C_j$ denotes the set of nodes assigned to the j-th community. The set of all structural community centers $U = \{ u_1, u_2, \dots , u_k \}$ forms a compact representation of the global community structure in the graph. In community detection, these centers serve as reference points, enabling the inference of node-community assignments by computing the similarity between node representations and each center.

### Definition 4

*Modularity.* Modularity Q is a widely used metric in network science for quantifying the quality of a given community partition by measuring its deviation from a random connectivity null model with the same degree distribution. Its mathematical definition is as follows:

3 $$\begin{aligned} {Q = \frac{1}{2M} \sum _{i=1}^{n} \sum _{j=1}^{n} \left( a_{ij} - \frac{d_i d_j}{2M} \right) \delta (c_i, c_j)} \end{aligned}$$

where $M$ represents the total number of edges in the network, $a_{ij}$ is an element of the adjacency matrix A, and $d_i$ denotes the degree of node $v_i$. The term $\frac{d_i d_j}{2M}$ indicates the expected number of edges between nodes $v_i$ and $v_j$. The variable $c_i$ represents the community label of node $v_i$, and $\delta (\cdot )$ is the Kronecker delta function, which equals 1 if two nodes belong to the same community and 0 otherwise. Today, modularity maximization is often directly employed as the optimization objective in community detection algorithms.

## Methods

**Fig. 1:**
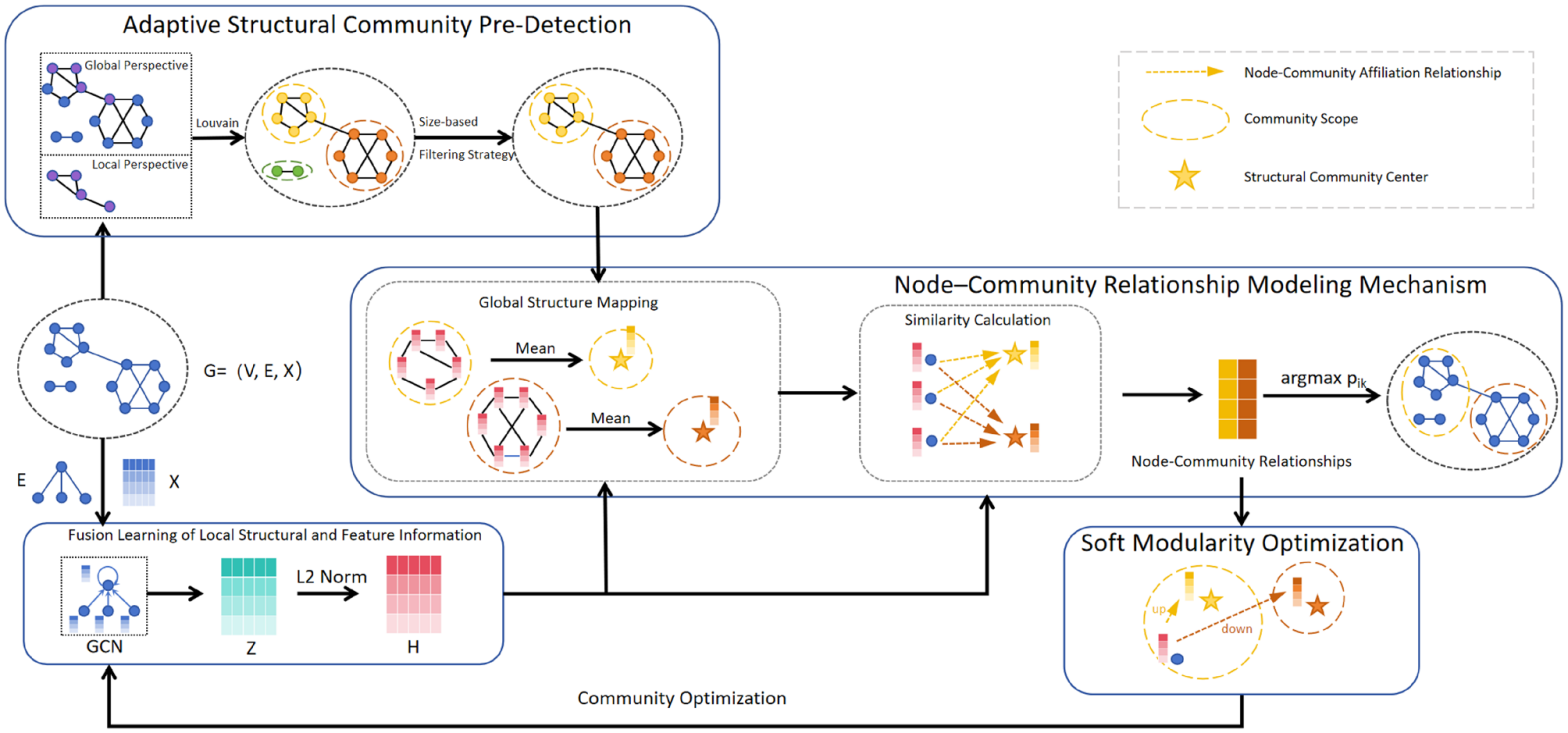
Model framework.

This section outlines the framework of the proposed method, as illustrated in Fig. 1. First, in the global structure extraction phase, the Louvain algorithm combined with a size-based filtering strategy is employed to adaptively identify structural communities with meaningful global topology, while effectively filtering out noisy clusters. This yields reliable global structural guidance. Second, a lightweight single-layer GCN is utilized to integrate node attributes and local structural information, generating low-dimensional embeddings that preserve essential relational patterns. Subsequently, through a mean aggregation operation, each structural community is mapped to a structural center within the same embedding space. A fine-grained node-community relational matrix is then constructed based on the similarity between node embeddings and these structural centers. Finally, a reconstructed soft modularity loss, derived from the modeled relationships, is optimized to directly refine the community membership associations among closely connected nodes, thereby deeply excavating the latent community structure.

### Adaptive structural community pre-detection based on global structure

In real-world networks, structure can be analyzed from both local and global perspectives. The global structural perspective helps capture the overall distribution pattern of communities but is often sensitive to noisy clusters. Conversely, the local structural perspective effectively identifies high-connectivity patterns among nearby nodes but is susceptible to interference from inter-community connections. As shown in the top-right corner of Fig. 1, the differences between the two are clearly noticeable.

To overcome the limitations of the local perspective and achieve adaptive community discovery, this paper proposes a global structure-guided approach for adaptive community identification and high-quality global structure extraction. The core idea is to leverage the macro-level distribution information embedded in the global network structure to provide boundary constraints and validation for partitioning locally dense regions, thereby dynamically guiding the delineation of community boundaries. The Louvain algorithm accomplishes adaptive community detection through iterative optimization of the network topology, continuously merging adjacent nodes and communities. Under the constraint of modularity, this method ensures both clear separation between communities and high cohesion within them, aligning well with our design principles of adaptive partitioning and global structure discovery.

Using the Louvain algorithm, we first extract preliminary structural communities $C=\{c_1,c_2,c_3,\ldots ,c_t \}$, with the number of communities denoted as $t$. Although these communities preserve the overall structural information of the graph, many of them are small in size and isolated, contributing little to the global structure and potentially introducing noise. To address this, we propose an adaptive size-based filtering mechanism. The threshold $T$ is determined by calculating the mean $\mu$ and standard deviation $\sigma$ of community sizes, and only communities exceeding this threshold are retained. The threshold $T$ is calculated as follows:

4 $$\begin{aligned} & \mu =\frac{n}{t} \end{aligned}$$

5 $$\begin{aligned} & \sigma = \sqrt{\frac{ {\textstyle \sum _{i=1}^{t}(\left| c_i \right| -\mu )^{2} } }{t} } \end{aligned}$$

6 $$\begin{aligned} & T=\mu +0.5\sigma \end{aligned}$$

Finally, the structural communities that satisfy the condition of having a community node count $|c_i |\ge T$ are denoted as $S=\{s_1,s_2,s_3,\ldots ,s_k \}$, where $k$ represents the number of structural communities that meet the requirements.

The threshold defined using the mean and standard deviation can automatically adapt to the size of the dataset without manual tuning. When community sizes vary greatly, the mean may decrease; however, the standard deviation will increase substantially due to the extreme differences. This effect raises the threshold, ensuring the retention of medium- and large-sized core communities that are more representative of the overall structure, thereby providing a stable and high-quality structural foundation.

### Fusion learning of local structural information and feature information

In attributed graphs, node attributes provide crucial feature information for community detection, effectively compensating for the limitations of relying solely on topological structure. Specifically, attribute features can transcend the constraints of topological connectivity, enabling nodes that are not directly connected but share similar attributes to form meaningful clusters in the feature space. This offers important clues for resolving the ambiguity in community assignments for nodes located at community boundaries.

Original node attributes typically contain only node-specific features and lack the capacity to capture relationships between related nodes, making it challenging to identify potentially similar nodes. To address this limitation, we employ GCNs to explicitly integrate node features with local structural information. GCNs utilize a symmetrically normalized adjacency matrix with self-connections, ensuring numerical stability during aggregation and mitigating the influence of node degree disparities. Through this aggregation process, GCNs combine each node’s features with those of its neighbors, enhance representational power and discriminative ability via nonlinear activation functions, and produce low-dimensional embeddings $Z\in \mathbb {R}^{n\times d'}$ that jointly encode attribute information and local structural patterns, thereby providing a robust foundation for subsequent community partitioning.

L2 normalization achieves feature scaling by dividing each vector by its Euclidean norm, thereby eliminating the influence of scale variation on similarity calculations. To this end, we apply L2 normalization to the node representations $Z$ learned by the GCN, obtaining the normalized embedding vectors $H\in \mathbb {R}^{n\times d'}$. The final node representation learning process is formulated as follows:

7 $$\begin{aligned} {H = \text {L2Norm}(\text {GCN}(X, A))} \end{aligned}$$

Normalized vectors exhibit geometric relationships determined solely by their directions, independent of their magnitudes. This property not only enhances computational efficiency but also ensures gradient stability during propagation.

### Node-community relationship modeling mechanism

We propose a multi-source information fusion mechanism for node-community relationship modeling, aiming to reconcile structural information and node features within a unified perspective, thereby directly capturing and revealing associations between nodes and communities. The core of this mechanism lies in mapping global structure, local topology, and node attributes into a unified embedding space, achieving alignment and integration of heterogeneous information sources. Within this space, soft membership relationships between nodes and communities are directly modeled, effectively leveraging the complementary advantages of multi-source information.

In this mechanism, the first step involves mapping global structural information into the continuous embedding space. Using the high-quality structural communities S with reference to the node representation matrix H, we compute a center matrix $U\in \mathbb {R}^{k\times d'}$, where the representation of the j-th community $s_j$ is derived by averaging the representations of all its member nodes:

8 $$\begin{aligned} u_j = \frac{ {\textstyle \sum _{h_i \in s_j}}h_i }{|s_j|} \end{aligned}$$

where $|s_j |$ represents the number of nodes in the j-th community, and $h_i$ represents the representation of j-th node belonging to the $s_j$ community. This operation materializes the abstract community structure $s_j$ into a point $u_j$, effectively mapping global structural information into a continuous vector space and laying the foundation for subsequent relationship modeling.

Following the global structure mapping, the second step infers the relationships between nodes and communities within the same space. Specifically, we quantify the association strength between a node and a community by measuring the similarity between the node representation $h_i$ and each structural community center $u_j$. Thanks to the normalization of the feature representations, using cosine similarity to accurately measure the similarity between node $h_i$ and structural center $u_j$ in the embedding space is equivalent to performing a vector dot product operation.

9 $$\begin{aligned} sim(h_i,u_j)=\frac{h_i\cdot u_j}{\left\| h_i\left\| \right\| u_j\right\| } =h_i\cdot u_j \end{aligned}$$

To make this similarity more intuitively reflect node-to-community assignments, the similarities are normalized using a Softmax function, yielding a node-community affiliation matrix $P\in \mathbb {R}^{n\times k}$,

10 $$\begin{aligned} p_{ij} = \frac{exp(-\delta \cdot sim(h_i,u_j))}{ {\textstyle \sum _{j=1}^{k}} exp(-\delta \cdot sim(h_i,u_j))} \end{aligned}$$

where $\delta$ is a temperature hyperparameter that controls the sharpness of the community distribution. By integrating global structure, local topology, and node attributes within a unified embedding space, this mechanism effectively leverages the complementary strengths of multi-source information. It not only offers more accurate membership determination for nodes at structural boundaries but also identifies and associates topologically disconnected nodes with high attribute similarity, ultimately enhancing the accuracy and robustness of community detection. Finally, the community to which a node belongs is determined according to the principle of maximum membership probability,

11 $$\begin{aligned} y_i=\underset{j}{argmax}~p_{ij} \end{aligned}$$

### Soft modularity-based community optimization

Modularity serves as a core metric for evaluating the quality of network community partitions, and its optimization process directly determines the rationality of community discovery. Traditional modularity functions rely on hard assignments, using discrete indicator functions to determine whether nodes belong to the same community. This approach fails to capture the strength of node-to-community affiliations and lacks flexibility in handling boundary nodes. To address this limitation, this study proposes a soft modularity function that incorporates a node-community affiliation probability matrix. This matrix transforms discrete community assignments into continuous probabilistic representations, enabling fine-grained optimization of node-community membership. By replacing the hard-assignment indicator function in traditional modularity with an inner product form of affiliation probabilities, we derive the soft modularity function $Q'$,

12 $$\begin{aligned} Q'=\frac{1}{2M}\sum _{ij} \sum _{m}^{k} (a_{ij}-\frac{d_id_j}{2M} )p_{im}p_{jm} \end{aligned}$$

where $a_{ij}$ is an element of the adjacency matrix A, $d_i=\sum _{j} a_{ij}$ denotes the degree of node $v_i$, and $p_{im}$ represents the affiliation probability of node i to community m. This design ensures that a node’s contribution to a community is proportional to its affiliation probability, allowing nodes to participate in the structural optimization of multiple communities through soft assignments. To simplify computation and improve optimization efficiency, the soft modularity function is converted into matrix form:

13 $$\begin{aligned} & B = A-\frac{dd^{T}}{2M} \end{aligned}$$

14 $$\begin{aligned} & Q' = \frac{1}{2M} tr[P^{T}BP] \end{aligned}$$

where $B$ denotes the modularity matrix, and $tr()$ denotes the trace of a matrix. This transformation not only reduces computational complexity but, more importantly, facilitates subsequent gradient-based optimization, enabling the modularity maximization process to be embedded into an end-to-end neural network training framework. The final loss function is:

15 $$\begin{aligned} L=-\alpha Q' \end{aligned}$$

$\alpha$ is the scaling factor. During the optimization process, the magnitude of a single loss value affects the gradient’s variation. Excessive changes in gradient magnitude can lead to an unstable training process. By setting an appropriate loss scaling factor $\alpha$, the loss values can be scaled to enhance the stability of the optimization, ensuring a smoother optimization process and preventing the algorithm from getting stuck in local optima.

Thus, unlike contrastive learning methods that rely on selecting positive and negative samples to make closely connected nodes more similar in the feature space, our approach leverages the node’s local structural connections, global structural position, and feature information to discover effective community-level information that enhances the correlation between nodes and their communities. This means that nodes with close connections within the same community will become increasingly similar. The specific process of the proposed method is shown in Algorithm 1.

### Are contrastive learning strategies necessary

In recent years, a large number of community detection methods have been developed that optimize node representations by incorporating contrastive learning strategies, thereby improving clustering performance. These methods typically construct positive and negative sample pairs based on structural proximity or semantic similarity, and enhance the separability of different communities in the embedding space by maximizing the consistency of positive sample pair representations and minimizing the consistency of negative sample pair representations.

In this study, we attempt to integrate three representative contrast strategies into the proposed method: contrast loss designs from CommDGI^21^, SupCon^40^, and MAGI^31^. In CommDGI, nodes within the same community as the current center are selected as positive samples, while nodes drawn from the nearest different community are used as negative samples. For SupCon and MAGI, the positive samples are defined as first-order neighbors within the same community, and the negative samples are nodes drawn from the nearest different community. This sampling scheme pulls together representations of nodes from the same class and pushes apart those from different classes in the embedding space, thereby learning more discriminative embeddings.

The CommDGI contrastive loss is computed as follows:

16 $$\begin{aligned} L_{CommDGI} = -\frac{1}{2n} {\textstyle \sum _{i=1}^{k}[ {\textstyle \sum _{V_+ \in M_+} \textrm{log} D(h_{v_+},u_i)+ {\textstyle \sum _{v_-} \textrm{log} (1-D(h_{v_-},u_i)) } } ] } \end{aligned}$$

where $M_+$ denotes the positive sample set, and $v_+$ represents a positive sample node. Similarly, $M_-$ represents the negative sample set, and $v_-$ represents a negative sample node. $D()$ denotes a discriminator, computed by applying a sigmoid function to the dot product of two vectors.

The SupCon contrastive loss is computed as follows:

17 $$\begin{aligned} L_{SupCon} = -\frac{1}{n} {\textstyle \sum _{i=1}^{n} \textrm{log}\left\{ \frac{1}{|M_+|} {\textstyle \sum _{v_{+} \in M_+}\frac{\textrm{exp} (h_{v_+}\cdot h_i)}{ {\textstyle \sum _{v \ne i} }\textrm{exp}(h_v\cdot h_i) } } \right\} } \end{aligned}$$

The MAGI contrastive loss is computed as follows:

18 $$\begin{aligned} L_{MAGI}=- {\textstyle \sum _{v_+\in M_+}}\textrm{log}\frac{\textrm{log}(z_i \cdot z_{v_+}/\tau )}{\sum _{v_+\in M_+} \textrm{exp}(z_i \cdot z_{v_+}/\tau ) + \sum _{v_-\in M_-}\textrm{exp}(z_i \cdot z_{v_-}/\tau )} \end{aligned}$$

Finally, all the aforementioned contrastive losses are collectively denoted as $L_{contarst}$, and incorporated into the total loss function as:

19 $$\begin{aligned} L=-\alpha Q'+\beta L_{contrast} \end{aligned}$$

By introducing different contrastive losses within the same framework, we can systematically evaluate the performance gains brought by contrastive strategies to our method and further verify the effectiveness of our model in the absence of such strategies. This design not only helps quantify the contribution of each contrastive strategy but also reveals the advantages of our model in simplifying the training process, reducing computational complexity, and enhancing generalization capability.

**Algorithm 1:**
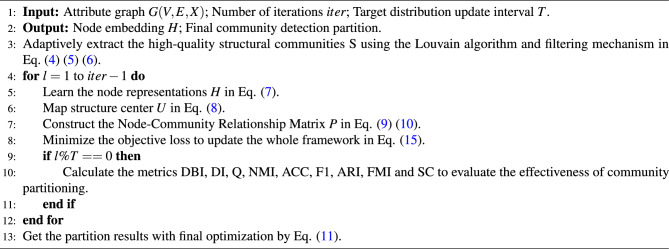
Community detection algorithm

## Experimentation

The experiments were conducted on a computer with an Intel i9 processor, 128GB of RAM, and the Windows 11 operating system, using a Python 3.8 environment for programming and computation.

### Datasets

The datasets used in our experiments can be categorized as follows:

Citation Networks^41^: Including Citeseer, Cora, and Pubmed, where nodes represent publications, edges represent citation relationships, node features are either bag-of-words or TF-IDF features, and labels correspond to publication topics.

Co-authorship Networks: ACM^42^ is a paper network where edges indicate shared authorship, while CoCS is a scholar network where edges represent co-authorship. Node features for both are bag-of-words from keywords, and labels denote research fields or subject categories, respectively.

Product Co-purchasing Networks: Amazon-Computers (Amac) and Amazon-Photo (Amap)^43^ contain computers and photography-related products, respectively, with edges indicating frequent co-purchase. Features are derived from review bag-of-words, and labels are product categories. Electronics-Photo (Ele-photo)^44^ is a network of electronics products based on co-purchase or co-view relationships.

Social Networks: Film^45^ is an actor network where edges denote co-appearance on the same Wikipedia page; UAT^46^ is an airport network where edges represent commercial flight routes. Labels indicate actor genres and passenger traffic levels, respectively.

The dataset is processed and provided by^47^, with a detailed description of the dataset shown in Table 2.

**Table 2:** Detailed description of the dataset.

Dataset	Nodes	Edges	Features	Communities
Acm	3025	13128	1870	3
Amac	7650	245861	767	10
Amap	13752	119081	745	8
Citeseer	3327	4552	3703	6
Cocs	18333	**81894**	6805	15
Cora	2708	5278	1433	7
Film	7600	15009	932	5
Pubmed	19717	44324	500	3
Uat	1190	13599	239	4
Ele-photo	48362	500928	384	12

### Comparison models

This paper selects eight representative community detection algorithms for comparison to comprehensively evaluate the performance of the proposed method. These algorithms cover three main paradigms: attribute-based, structure-based, and methods that integrate both attributes and structure.

K-means^15^ is a classic attribute-based clustering algorithm. It partitions nodes solely based on the distribution of their feature vectors in the latent space, serving as a baseline for attribute-only clustering.

Louvain^38^ is a heuristic structure-based algorithm. It iteratively merges nodes to maximize modularity, serving as a benchmark for pure structural methods.

Six baseline algorithms that integrate attributes and structure are selected:

CommDGI^21^ learns node embeddings by maximizing the mutual information between local node representations and a global graph summary.

DGCluster^30^ transforms modularity maximization into a differentiable loss function, enabling end-to-end joint optimization with GNN-based representation learning.

DCGL^23^ employs a pseudo-siamese network architecture to extract features from structural and attribute perspectives separately, enhancing the representations through cross-view contrastive learning.

MAGI^31^ uses the modularity matrix as an anchor for contrastive learning, capturing high-order structural similarity without requiring graph augmentation.

MGCN^39^ designs multi-hop graph convolution to adaptively fuse information from higher-order neighborhoods, learning more comprehensive node representations.

CPGCL^26^ jointly learns node representations and soft community assignments, dynamically refining sample pairs in contrastive learning to alleviate the false negative problem.

### Evaluation metrics and parameter settings

In this experiment, the community detection task in attribute graphs will be the main focus, and the performance of all community detection methods will be compared. To evaluate the quality of the predicted communities, we use eight evaluation metrics: DBI, Q, NMI, ACC, F1-score, ARI, FMI and SC to assess the effectiveness of the community detection results. The DBI metric primarily measures the similarity and separation between the detected communities, aiming to make communities more compact internally and more separated from each other. A smaller DBI value is preferable. For the other metrics, higher values are better, that is

20 $$\begin{aligned} DBI = \frac{1}{k} {\textstyle \sum _{i=1}^{k}}max_{j\ne i}(\frac{avg(c_i)+avg(c_j)}{d_{cen}(u_i+u_j)} ) \end{aligned}$$

where k represents the number of communities, $avg(c_i )$ represents the average distance of all nodes in the i-th community to its center, and $d_{cen} (u_i+u_j)$ represents the distance between the centers of the i-th and j-th communities.

The NMI metric is normalized based on the concept of mutual information from information theory, and is used to measure the similarity between the community detection results and the ground truth, that is

21 $$\begin{aligned} & MI(C,G)=\sum _{c\in C} \sum _{g \in G} P(c,g)log\frac{P(c,g)}{P(c)P(g)} \end{aligned}$$

22 $$\begin{aligned} & H(G)=- \sum _{g\in G} P(g)logP(g) \end{aligned}$$

23 $$\begin{aligned} & NMI(C,G)=\frac{MI(C,G)}{\sqrt{H(C)\cdot H(G)} } \end{aligned}$$

where $G$ represents the ground truth, and $C$ represents the community detection results. $P(c,g)$ denotes the joint probability distribution of a node being in both the true community g and the detected community c. $P(g)$ represents the probability of a node being in the true community g. $MI()$ represents mutual information, and $H()$ represents entropy.

The ACC metric is used to measure the consistency between the community detection results and the ground truth, that is

24 $$\begin{aligned} ACC=\frac{1}{n} {\textstyle \sum _{i=1}^{n}}\rho (y_i,map(\hat{y}_i )) \end{aligned}$$

where $y_i$ represents the true community label of i-th node, and $\hat{y}_i$ represents the community detection label of the node. $\rho$ is an indicator function that takes the value 1 if the true label and the detected label are the same, and 0 otherwise. $map(\hat{y}_i )$ denotes the mapping of the detection label of node *i* to the true label.

The F1-score provides a comprehensive evaluation of the model’s precision and recall, measuring the consistency between the communities detected by the algorithm and the true communities, that is

25 $$\begin{aligned} & F1=2\cdot \frac{Precision\cdot Recall}{Precision+ Recall} \end{aligned}$$

26 $$\begin{aligned} & Precision=\frac{TP}{TP+FP} \end{aligned}$$

27 $$\begin{aligned} & Recall=\frac{TP}{TP+FN} \end{aligned}$$

where *TP* represents the number of nodes predicted to belong to community *c* and actually belong to *c*, *FP* represents the number of nodes predicted to belong to community *c* but do not belong to *c*, *FN* represents the number of nodes that actually belong to community *c* but are predicted not to belong to *c*.

The ARI is a metric used to measure the similarity between detection results and true labels, that is

28 $$\begin{aligned} & RI=2\cdot \frac{TP+TN}{n(n-1)} \end{aligned}$$

29 $$\begin{aligned} & ARI=\frac{RI-E[RI]}{max(RI)-E[RI]} \end{aligned}$$

where *TN* represents the number of nodes that do not actually belong to community *c* and are predicted not to belong to *c*, and *E*[] denotes the expected value.

The FMI metric measures the geometric mean of precision and recall for community detection results, balancing the trade-off between false positives and false negatives, that is

30 $$\begin{aligned} \textrm{FMI} = \frac{\textrm{TP}}{\sqrt{(\textrm{TP}+\textrm{FP})\cdot (\textrm{TP}+\textrm{FN})}} = \sqrt{Precision \cdot Recall} \end{aligned}$$

The SC metric evaluates the compactness within communities and the separation between communities, that is

31 $$\begin{aligned} & SC=\frac{1}{n}\sum _{i=1}^{n} SC(x_i) \end{aligned}$$

32 $$\begin{aligned} & SC(x)=\frac{b(x)-a(x)}{\max \!\bigl \{a(x),\,b(x)\bigr \}} \end{aligned}$$

33 $$\begin{aligned} & a(x)=\frac{1}{|C_x|-1}\sum _{\begin{array}{c} x'\in C_x \end{array}} d(x,x') \end{aligned}$$

34 $$\begin{aligned} & b(x)=\min _{C_j\ne C_x}\Bigl \{\frac{1}{|C_j|}\sum _{x'\in C_j} d(x,x')\Bigr \} \end{aligned}$$

where *SC*(*x*) is the Silhouette Score of node *x*. *a*(*x*) is the mean intra-cluster distance of node *x*, and *b*(*x*) is the minimum mean distance from *x* to any other cluster. SC ranges from $-1$ to 1, with higher values indicating more coherent and well-separated clusters.

In our work, a single-layer convolutional network is used for node representation learning. The hyperparameter $\delta$ is set to 30, and the loss coefficient $\alpha$ is set to 0.001. The Adam optimizer is used for 300 iterations of model training, with a learning rate of 0.001 and weight decay set to 0.005. In all experiments, the dimension of node representations is fixed at 512. For comparison experiments, the number of communities to be specified is set to the number of communities detected in this experiment. All other parameters for baseline methods follow their original papers to ensure optimal performance. For example, CommDGI uses a learning rate of 0.001 for 500 iterations; DGCluster uses 0.001 for 300 iterations; DCGL uses 0.001 for 300 iterations; MAGI uses 0.0005 for 400 iterations; MGCN uses 0.003 for 700 iterations; and CPGCL uses 0.0007 for 600 iterations.

**Table 3:** The performance comparison of different community detection algorithms

Dataset	Metrics	K-means	Louvain	CommDGI	DGCluster	DCGL	MAGI	MGCN	CPGCL	Our
Cora	Min DBI	–	–	1.554865	0.958007	1.916085	2.142677	**0.457259**	3.370206	0.458970
	Max Q	0.216550	**0.783224**	0.685146	0.752858	0.288298	0.722241	0.211403	0.480478	0.765320
	Max NMI	0.233923	0.457330	0.518370	0.465627	0.230544	**0.591830**	0.225887	0.233233	0.561596
	Max ACC	0.364845	0.519202	0.666913	0.274003	0.408789	**0.691285**	0.361521	0.388848	0.669129
	Max F1	0.392400	0.069800	0.546800	0.231300	0.325500	**0.703900**	0.296000	0.377249	0.663200
	Max ARI	0.123668	0.310949	0.444888	0.154347	0.110080	**0.560715**	0.082515	0.156608	0.469243
	Max FMI	0.285960	0.419639	0.560501	0.297193	0.409037	**0.579507**	0.420040	0.287833	0.562167
	Max SC	0.030930	0.095201	0.434185	0.561923	0.046621	0.478210	0.285998	0.067724	**0.858014**
Citeseer	Min DBI	–	–	1.718555	0.682854	1.712106	2.810201	**0.458751**	4.929488	0.583929
	Max Q	0.342458	0.783224	0.718006	0.813808	0.286117	0.794073	0.177122	0.462403	**0.822334**
	Max NMI	0.231280	**0.457330**	0.353227	0.351596	0.138506	0.341371	0.095831	0.094860	0.385114
	Max ACC	0.421100	0.519202	0.545537	0.122633	0.360986	0.420800	0.235047	0.276225	**0.559663**
	Max F1	0.380200	0.069800	0.438600	0.117700	0.289200	0.446200	0.188400	0.258641	**0.504300**
	Max ARI	0.172758	0.310949	0.312169	0.057623	0.087879	0.217544	0.004307	0.072031	**0.356296**
	Max FMI	0.290782	0.279003	0.448738	0.162507	0.416197	0.341115	0.386031	0.208526	**0.459963**
	Max SC	0.014913	0.047396	0.420256	0.616554	0.057491	0.289657	0.164237	0.011400	**0.814350**
Acm	Min DBI	–	–	0.626647	0.829712	1.162722	1.828684	**0.261183**	3.167038	0.684481
	Max Q	0.179559	0.783224	0.585480	**0.793614**	0.162135	0.732835	0.427613	0.655669	0.745318
	Max NMI	0.287808	0.457330	**0.648341**	0.382356	0.214327	0.431209	0.069817	0.154253	0.620495
	Max ACC	0.351074	0.519202	**0.875041**	0.270413	0.525289	0.338843	0.333223	0.303471	0.844300
	Max F1	0.411000	0.069800	**0.742500**	0.249200	0.382500	0.446000	0.289200	0.342566	0.670900
	Max ARI	0.193426	0.310949	**0.693615**	0.165469	0.177992	0.256714	0.000088	0.113481	0.692292
	Max FMI	0.415971	0.320982	**0.825851**	0.357503	0.575100	0.479568	0.523107	0.308405	0.788906
	Max SC	0.014176	0.108579	0.540433	0.552963	0.010168	0.305496	0.512783	0.035922	**0.798375**
Amap	Min DBI	–	–	0.539270	1.272281	1.255727	0.760000	0.486686	NAN	**0.377126**
	Max Q	0.080476	**0.783224**	0.346104	0.680452	0.435044	0.711067	0.240120	NAN	0.670459
	Max NMI	0.117092	0.457330	0.220800	**0.695787**	0.514492	0.674966	0.169413	NAN	0.646764
	Max ACC	0.291503	0.519202	0.389150	0.661961	0.669412	**0.786144**	0.290588	NAN	0.685882
	Max F1	0.284700	0.069800	0.310100	0.452600	0.650500	**0.776100**	0.225300	NAN	0.684800
	Max ARI	0.048474	0.310949	0.085440	0.547796	0.434472	**0.583538**	0.015830	NAN	0.506579
	Max FMI	0.220878	0.505307	0.437640	**0.633039**	0.495231	0.601123	0.332593	NAN	0.607000
	Max SC	0.167072	0.322381	0.847948	0.764490	0.194227	0.730850	0.591428	NAN	**0.899968**
Uat	Min DBI	-	-	1.650489	0.995580	0.609719	2.086179	0.542170	2.117715	**0.453726**
	Max Q	0.000959	**0.783224**	0.219029	0.268062	0.049000	0.229935	0.113806	0.174967	0.280535
	Max NMI	0.214282	0.116344	0.261743	0.194440	**0.268799**	0.135860	0.266431	0.097880	0.248141
	Max ACC	0.430252	0.356303	**0.556303**	0.234454	0.468908	0.457983	0.336134	0.380672	0.547059
	Max F1	0.451700	0.075200	0.570600	0.225900	0.404000	0.444900	0.438200	0.353601	**0.575400**
	Max ARI	0.144351	0.087934	0.248691	0.086840	**0.404000**	0.118683	0.119921	0.070222	0.243589
	Max FMI	0.427409	0.412096	0.447597	0.224049	**0.484028**	0.369455	0.475867	0.387757	0.476756
	Max SC	0.312638	0.218247	0.609281	0.515990	0.574159	0.419987	0.470890	0.408284	**0.648977**
Ele-Photo	Min DBI	–	–	2.274522	1.358343	N/A	0.883479	OM	OM	**0.730426**
	Max Q	0.328653	**0.797354**	0.678545	0.604822	N/A	0.682104	OM	OM	0.739465
	Max NMI	0.244915	0.425891	0.450860	0.381566	N/A	0.435163	OM	OM	**0.453136**
	Max ACC	0.322939	0.445763	0.519189	0.272177	N/A	0.460672	OM	OM	**0.533353**
	Max F1	0.276600	0.036300	0.367200	0.179200	N/A	**0.502600**	OM	OM	0.487800
	Max ARI	0.089260	0.155509	**0.258453**	0.070701	N/A	0.206226	OM	OM	0.240034
	Max FMI	0.229346	0.287502	**0.395542**	0.192001	N/A	0.342707	OM	OM	0.370855
	Max SC	0.043629	0.356345	0.375267	0.423660	N/A	0.641512	OM	OM	**0.696875**

“-” indicates that the metric is not applicable to the algorithm, “OM” indicates an out-of-memory error occurred, “N/A” indicates the algorithm’s runtime exceeded five days, and “NAN” indicates the algorithm encountered a NAN error. Bold values represent the best results, and underlined values represent the runner-up results

### Experiment result

This paper compares the performance of seven community detection methods for the community detection task. Table 3 summarizes the results of the community performance comparison across different algorithms. Bold numbers indicate the best performance, while underlined numbers indicate the runner-up performance.

As shown in Table 3, the proposed algorithm achieves the best or runner-up performance in most evaluation metrics across different datasets. Compared to other deep learning-based methods, our approach incorporates global, local structural, and feature information for community detection, enhancing modularity loss by leveraging community membership probabilities derived from these three information sources. By maximizing the improved modularity loss, the algorithm effectively uncovers the community memberships of nodes, leading to strong experimental results in the community detection task.

### Comprehensive algorithm evaluation and statistical validation

*Experiment 1: Wilcoxon signed-rank test for algorithm comparison*

The Wilcoxon signed-rank test^48^ is a non-parametric statistical method used to compare paired or related samples. This study adopted a significance level of 0.05 to assess the differences between the proposed algorithm and comparative algorithms. The results are recorded in Table  4. The terms R+ and R- represent the sum of ranks where the proposed algorithm performed superior or inferior to its competing algorithms, respectively. A p-value below 0.05 indicates a statistically significant difference between the proposed algorithm and the compared algorithm. Specifically, when the p-value is less than 0.05, it signifies a significant difference between the proposed algorithm and the comparative algorithm, which is highlighted in bold in the table.

Table 4 presents the Wilcoxon test results for the ACC, F1, ARI, and SC metrics of the algorithms. The data results demonstrate that the metrics of the proposed algorithm differ significantly from those of the comparison algorithms, thus validating its strong performance across the 10 datasets.

**Table 4:** Results of the Wilcoxon signed-rank test

Our vs.	ACC			F1			ARI			SC		
	R+	R-	*p*-value	R+	R-	*p*-value	R+	R-	*p*-value	R+	R-	*p*-value
K-means	53	2	**0.009344**	54	1	**0.00691**	52	3	**0.012515**	55	0	**0.005062**
Louvain	48	7	**0.036658**	55	0	**0.005062**	44	11	0.092601	55	0	**0.005062**
CommDGI	46	9	0.059336	51	4	**0.016605**	48	7	**0.036658**	50	5	**0.021824**
DGCluster	55	0	**0.005062**	55	0	**0.005062**	53	2	**0.009344**	55	0	**0.005062**
DCGL	21	0	**0.027708**	36	0	**0.011719**	24	12	0.400814	21	0	**0.027708**
MAGI	46	9	0.059336	39	16	0.241121	44	11	0.092601	55	0	**0.005062**
MGCN	36	0	**0.011719**	55	0	**0.005062**	53	2	**0.009344**	36	0	**0.011719**
CPGCL	21	0	**0.027708**	21	0	**0.027708**	21	0	**0.027708**	21	0	**0.027708**

Bold values indicate statistically significant differences (*p* < 0.05).

*Experiment 2: Comprehensive ranking experiment based on multi-criteria decision-making*

To comprehensively evaluate the overall performance of different algorithms, this study adopts the TOPSIS^49^ method as a multi-criteria decision-making (MCDM) approach^50^. Considering that the eight evaluation metrics used in this experiment are of diverse types and that some of them are highly correlated, the CRITIC method^51^ is employed to automatically compute objective weights, thereby avoiding the subjectivity of manual weight assignment.

The experimental procedure is as follows: first, missing values and special entries (e.g., OM, N/A) in the original evaluation metrics are processed, followed by orientation unification and normalization to eliminate differences in scale and direction; next, the CRITIC method is applied to determine the weights by jointly considering the discrimination power and information independence of each metric; finally, the TOPSIS method is used to calculate the closeness coefficient of each algorithm to the positive and negative ideal solutions, and the algorithms are ranked in descending order of the coefficient, yielding an objective and unified performance ranking in the multi-dimensional evaluation system.

As shown in Table 5, our method consistently captures the overall performance differences among algorithms across multiple real-world datasets. It achieves the top rank on most datasets, thereby demonstrating its superiority in terms of comprehensive performance across multiple evaluation metrics.

### Ablation experiments

*Experiment 1: Effectiveness analysis of the adaptive structural community extraction module*

The first ablation experiment aims to remove the Louvain-based adaptive structural community extraction module to verify the effectiveness of global structure information. Since the proposed method relies on global centroids during the fusion stage to compute node-community memberships, in the absence of Louvain, we directly perform K-means clustering on the learned node embedding matrix $H$, using the ground-truth number of communities $K_R$ as the number of clusters to generate global centroids. These centroids then replace the structural community centers extracted by Louvain in the subsequent steps, allowing us to evaluate the contribution and necessity of Louvain pre-detection to the quality of global structural information.

The experimental results are shown in Table 6. Specifically, the model incorporating global structural information outperforms the counterpart without global structural information across multiple evaluation metrics, indicating that global structural information plays a significant role in improving the accuracy and stability of community partitioning, thereby highlighting its necessity in the proposed method.

**Table 5:** Comprehensive performance ranking of different community detection methods based on MCDM.

		Kmeans	Louvain	CommDGI	DGCluster	DCGL	MAGI	MGCN	CPGCL	Our
Cora	Topsis score	0.1815	0.5131	0.7164	0.5515	0.3290	0.6856	0.4751	0.2225	**0.9387**
	Rank	9	5	2	4	7	3	6	8	1
Citeseer	Topsis score	0.4160	0.5444	0.7609	0.5341	0.4591	0.6187	0.4471	0.2304	**0.9346**
	Rank	8	4	2	5	6	3	7	9	1
Acm	Topsis score	0.2164	0.4909	0.8133	0.5610	0.3819	0.5177	0.4993	0.3270	**0.9080**
	Rank	9	6	2	3	7	4	5	8	1
Amap	Topsis score	0.1019	0.4939	0.4581	0.6469	0.5057	0.8236	0.3566	0.0000	**0.8864**
	Rank	8	5	6	3	4	2	7	9	1
Film	Topsis Score	0.4113	0.5057	0.5103	0.2829	0.5534	0.4109	0.5653	0.3883	**0.6225**
	Rank	6	5	4	9	3	7	2	8	1
Pubmed	Topsis score	0.6262	0.5372	0.5733	0.2688	0.0000	0.5083	0.4584	0.3557	**0.7189**
	Rank	2	4	3	8	9	5	6	7	1
Cocs	Topsis score	0.5866	0.3198	0.7030	0.4180	0.0000	0.4774	0.3614	0.0000	**0.9288**
	Rank	3	7	2	5	8	4	6	8	1
Amac	Topsis score	0.1776	0.3001	0.5332	0.5739	0.0000	0.6304	0.0000	0.0000	**0.7852**
	Rank	6	5	4	3	7	2	7	7	1
Uat	Topsis score	0.4162	0.4807	0.7473	0.4962	0.6199	0.4659	0.6064	0.3495	**0.8252**
	Rank	8	6	2	5	3	7	4	9	1
Ele-Photo	Topsis score	0.2069	0.5361	0.6487	0.3701	0.0000	0.8089	0.0000	0.0000	**0.9317**
	Rank	6	4	3	5	7	2	7	7	1

**Table 6:** Effectiveness analysis of the adaptive structural community extraction Module.

		Min DBI	Max Q	Max NMI	Max ACC	Max F1	Max ARI	Max FMI	Max SC
Cora	With global structure	**0.458282**	**0.765320**	**0.561161**	**0.673929**	**0.663000**	**0.472555**	0.562167	**0.858014**
	Without global structure	0.676832	0.681079	0.528015	**0.673929**	0.657000	0.451359	**0.566008**	0.708492
Acm	With global structure	**0.684481**	**0.745318**	**0.620495**	**0.844298**	0.670900	**0.692292**	**0.788906**	**0.798375**
	Without global structure	2.697249	0.470766	0.460258	0.639339	**0.740500**	0.428861	0.678737	0.269921
Amap	With global structure	0.377126	**0.670459**	**0.646764**	**0.685882**	**0.684800**	**0.506579**	**0.607000**	**0.899968**
	Without global structure	**0.242789**	0.461736	0.347316	0.452810	0.451600	0.233878	0.453156	0.834500
Uat	With global structure	0.774019	**0.280535**	**0.248141**	**0.547059**	**0.575400**	**0.243589**	0.476756	0.648977
	Without global structure	**0.723423**	0.241941	0.219666	0.512605	0.500600	0.196388	**0.499371**	**0.655903**

*Experiment 2: Effectiveness analysis of the contrastive strategy*

To evaluate the impact of different contrastive learning strategies on the performance of our method, we incorporated three representative contrastive losses–$L_{CommDGI}$, $L_{SupCon}$ and $L_{MAGI}$–into the same framework and compared them with a baseline model without contrastive optimization. All experiments were conducted on the same datasets (Cora, Acm, Amap, and Uat) to ensure comparability, with the contrastive loss coefficient $\beta$ tested at values of 1, 0.1, 0.01, and 0.001, and the best-performing setting ($\beta$=0.001) adopted for reporting results. Performance was quantitatively assessed using six metrics: DBI, Q, NMI, ACC, F1-score, and ARI.

The detailed results are presented in Tables 7, 8 and 9, where Table 7 compares the results with and without $L_{CommDGI}$, Table 8 compares the results with and without $L_{SupCon}$, and Table 9 compares the results with and without $L_{MAGI}$. These comparisons intuitively reveal the actual performance gains of different contrastive strategies and validate the effectiveness of our method in the absence of contrastive optimization.

**Table 7:** Experimental results with and without $L_{CommDGI}$ loss.

		Min DBI	Max Q	Max NMI	Max ACC	Max F1	Max ARI
Cora	With contrastive loss	0.459781	0.762369	0.559834	**0.681315**	0.655900	**0.489056**
	Without contrastive loss	**0.458282**	**0.765320**	**0.561161**	0.673929	**0.663000**	0.472555
Acm	With contrastive loss	**0.590283**	**0.746271**	0.597249	0.836364	0.662600	0.658855
	Without contrastive loss	0.684481	0.745318	**0.620495**	**0.844298**	**0.670900**	**0.692292**
Amap	With contrastive loss	**0.356660**	0.660752	0.645052	0.622745	0.654600	0.487767
	Without contrastive loss	0.377126	**0.670459**	**0.646764**	**0.685882**	**0.684800**	**0.506579**
Uat	With contrastive loss	**0.707185**	0.279933	0.246803	0.547059	**0.579100**	0.243487
	Without contrastive loss	0.774019	**0.280535**	**0.248141**	**0.547059**	0.575400	**0.243589**

**Table 8:** Experimental results with and without $L_{SupCon}$ loss.

		Min DBI	Max Q	Max NMI	Max ACC	Max F1	Max ARI
Cora	With contrastive loss	0.631657	0.721620	0.505175	0.579025	0.626200	0.384827
	Without contrastive loss	**0.458282**	**0.765320**	**0.561161**	**0.673929**	**0.663000**	**0.472555**
Acm	With contrastive loss	0.868040	**0.752136**	0.414174	0.628430	0.510600	0.421314
	Without contrastive loss	**0.684481**	0.745318	**0.620495**	**0.844298**	**0.670900**	**0.692292**
Amap	With contrastive loss	0.770020	0.650918	0.586971	0.597908	0.606000	0.449165
	Without contrastive loss	**0.377126**	**0.670459**	**0.646764**	**0.685882**	**0.684800**	**0.506579**
Uat	With contrastive loss	**0.643772**	0.218710	0.212367	0.527731	0.523000	0.197494
	Without contrastive loss	0.774019	**0.280535**	**0.248141**	**0.547059**	**0.575400**	**0.243589**

**Table 9:** Experimental results with and without $L_{MAGI}$ loss.

		Min DBI	Max Q	Max NMI	Max ACC	Max F1	Max ARI
Cora	With contrastive loss	0.501831	0.746360	0.544831	0.666913	0.638700	**0.488040**
	Without contrastive loss	**0.458970**	**0.765320**	**0.561596**	**0.669129**	**0.663200**	0.469243
Acm	With contrastive loss	0.916360	0.728609	0.516628	0.744463	0.553700	0.544837
	Without contrastive loss	**0.684481**	**0.745318**	**0.620495**	**0.844300**	**0.670900**	**0.692292**
Amap	With contrastive loss	0.457712	0.643710	0.608214	0.615294	0.623500	0.466393
	Without contrastive loss	**0.377126**	**0.670459**	**0.646764**	**0.685882**	**0.684800**	**0.506579**
Uat	With contrastive loss	**0.206296**	0.167113	0.227279	0.510924	0.491400	0.206899
	Without contrastive loss	0.807088	**0.280636**	**0.249595**	**0.546218**	**0.578200**	**0.242806**

### Parameter analysis

*Experiment 1: Sensitivity Analysis of the Threshold Filtering Mechanism*

This experiment analyzes the threshold filtering mechanism applied after Louvain-based community partitioning. To evaluate the sensitivity of this mechanism to threshold selection, we vary the standard deviation coefficient from 0.1 to 1.0 and systematically examine the impact of different threshold strategies on both the fidelity of global information and the final detection performance. This process helps verify the effectiveness and robustness of the filtering mechanism in balancing noise suppression with information preservation.

**Fig. 2:**
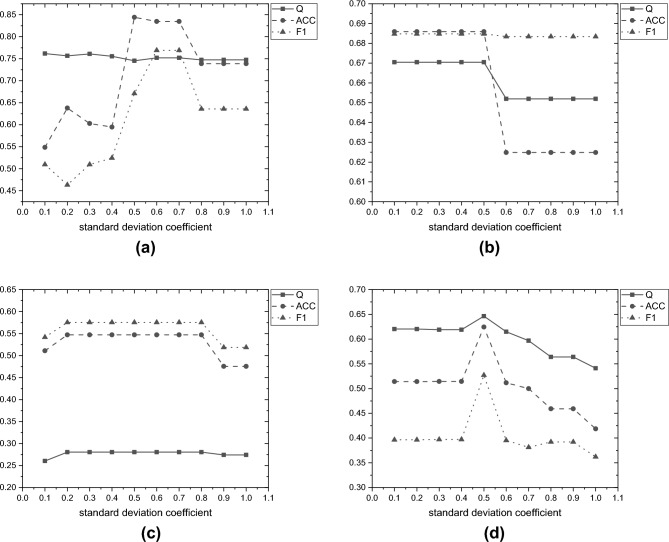
Scores of Q, ACC, and F1 metrics for the Acm, Amap, Uat, and Cocs datasets under different standard deviation coefficients.

We analyzed the trends of the Q, ACC, and F1 metrics on the Acm, Amap, Uat, and Cocs datasets. As shown in Fig. 2, when the standard deviation coefficient is in the range of 0.4-0.6, most metrics achieve both superior and stable performance. In particular, a coefficient of 0.5 achieves a good balance among Q, ACC, and F1.

**Fig. 3:**
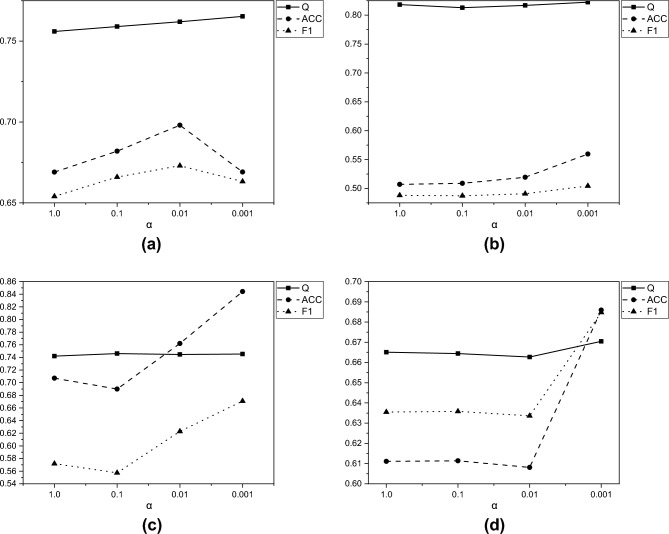
Scores of Q, ACC, and F1 metrics for the Cora, Citeseer, Acm, and Amap datasets under different $\alpha$ values.

*Experiment 2: Sensitivity Analysis of the* $\alpha$*Parameter*

In this experiment, we investigate the impact of different values of $\alpha$ on the performance of the experiment across various datasets. Specifically, we set $\alpha$ = 1.0, 0.1, 0.01, 0.001 and perform experiments on the Cora, Citeseer, ACM, and AMAP datasets, observing the Q, NMI, and ACC metrics. The experimental results shown in Fig. 3 demonstrate that when $\alpha$ is set to 0.001, the performance is optimal. This is because a smaller modularity loss helps mitigate the problem of modularity optimization getting stuck in local optima, preventing overfitting caused by forcing the algorithm to rigidly determine community memberships. A more comprehensive analysis of the relationships between all communities allows for better extraction of community membership information.

### Depth sensitivity analysis of GCN

To further investigate whether increasing the number of GCN layers can enhance the model’s ability to capture deep local features and attribute associations, we designed a depth sensitivity experiment using single-layer, two-layer, and three-layer GCN encoders. All parameters other than the number of layers were kept consistent with the baseline to ensure result comparability.

The experimental results, as shown in Table 10, indicate that excessively deep GCNs suffer from over-smoothing, leading to a decline in accuracy. Since the proposed method already incorporates rich global structural information, a single-layer GCN is sufficient to capture the necessary local structural features, thereby achieving a better balance between performance and computational efficiency.

**Table 10:** Performance of GCNs with different numbers of layers in depth sensitivity Experiments.

		Min DBI	Max Q	Max NMI	Max ACC	Max F1	Max ARI	Max FMI	Max SC	Topsis Score
Citeseer	single-layer GCN	0.583929	**0.822334**	**0.385114**	0.559663	0.504300	**0.356296**	**0.459963**	0.814350	**0.515353**
	two-layer GCN	0.484069	0.818214	0.377790	0.599940	0.568800	0.345601	0.456540	**0.842465**	0.492008
	three-layer GCN	**0.444321**	0.816524	0.376340	**0.617072**	**0.605500**	0.336664	0.459849	0.792908	0.515290
Acm	single-layer GCN	0.684481	0.745318	**0.620495**	**0.844300**	**0.670900**	**0.692292**	**0.788906**	**0.798375**	**0.518493**
	two-layer GCN	0.589847	**0.752684**	0.474369	0.621157	0.524600	0.475670	0.626644	0.598632	0.507636
	three-layer GCN	**0.572061**	0.751419	0.440557	0.606281	0.599700	0.435782	0.595400	0.670572	0.502263
Amap	single-layer GCN	**0.377126**	**0.670459**	**0.646764**	**0.685882**	**0.684800**	**0.506579**	**0.607000**	**0.899968**	**0.791961**
	two-layer GCN	0.536609	0.661113	0.641394	0.608366	0.629600	0.486151	0.604802	0.751231	0.251217
	three-layer GCN	0.566904	0.659047	0.603202	0.599869	0.621300	0.468950	0.587254	0.692937	0.487811
Cocs	single-layer GCN	0.534169	**0.646346**	**0.528356**	**0.624284**	**0.527100**	**0.508426**	**0.586879**	**0.823781**	**0.681961**
	two-layer GCN	0.636651	0.614536	0.390344	0.522064	0.392900	0.382378	0.482495	0.544767	0.381217
	three-layer GCN	**0.479450**	0.591030	0.289053	0.442481	0.337100	0.315236	0.411786	0.717388	0.327811

### Runtime comparison

**Fig. 4:**
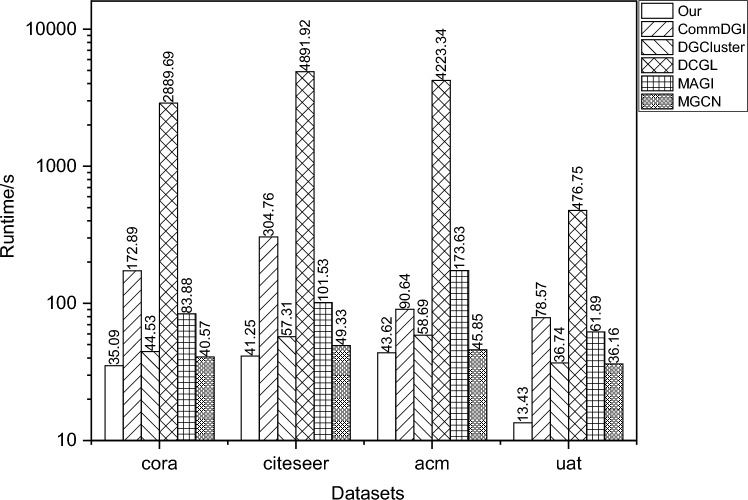
Runtime comparison of various algorithms on Cora, Citeseer, Acm, and Uat datasets.

In this experiment, the runtime comparison of various deep learning-based algorithms is conducted on four different datasets: Cora, Citeseer, Acm, and Uat. As shown in the experimental comparison in Fig. 4, our algorithm achieves the fastest runtime across different datasets. The reason for this is that the community detection framework designed in this paper employs the fast Louvain algorithm for pre-community detection to obtain global structural information, and uses a basic GCN to integrate local structural and feature information for learning node representations. These useful pieces of information are then fused to compute the community membership probabilities of nodes. Finally, modularity-based community optimization is performed using the membership probabilities to mine the community information, which is faster and more effective compared to other deep learning-based algorithms. Our community detection framework reduces the extra view generation for data augmentation, the construction of positive and negative samples for contrastive learning, and the joint optimization of multiple objectives, achieving rapid and effective detection with a simple yet efficient framework and optimization approach.

**Fig. 5:**
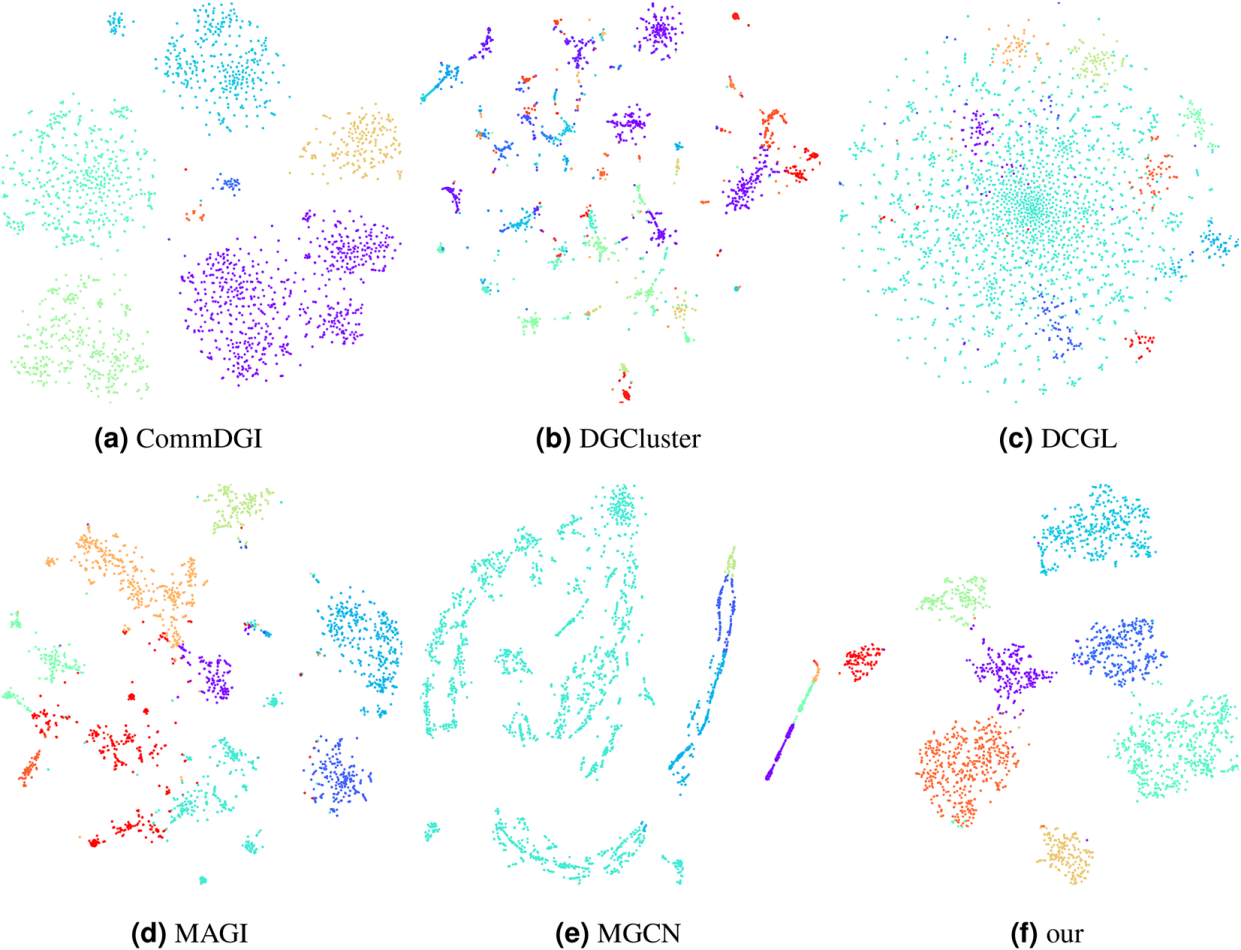
T-SNE visualization results on the Cora dataset.

### Visualization comparison of community detection results

To intuitively verify the effectiveness of the algorithm presented in this paper, we use the T-distributed stochastic neighbor embedding (T-SNE) algorithm^52^ to visualize the final node representations and community partition results in a two-dimensional space, as shown in Fig. 5. In this figure, we compare the community detection results after learning node representations based on the original features using K-means and deep learning on the Cora dataset. Our method more effectively distinguishes community differences and ensures community cohesion.

## Conclusion

In this study, we proposed a straightforward and effective approach for community detection. Our method features adaptive detection, identifying high-quality structural communities without needing a predefined number of communities. It also includes node-community relationship modeling, which integrates local topology and node attributes into a shared embedding space, allowing us to model the soft relationships between nodes and community centers. Additionally, we implement soft modularity reconstruction, which optimizes community partitioning in an end-to-end manner using the soft relational matrix, without relying on contrastive learning or data augmentation. Our approach offers a new perspective on the research landscape, hoping to inspire researchers to develop improved algorithms for community detection.

We validate the effectiveness of our proposed algorithm through comparative experiments using various metrics, including DBI, Q, NMI, ACC, F1, ARI, FMI, and SC. In future work, we plan to explore different community detection techniques and social network analysis methods, focusing on optimizing the acquisition of global structural information.

## Data Availability

The datasets generated during and/or analysed during the current study are available in the Github repository, https://github.com/wuanghoong/Less-is-More.git.
